# RNA-Seq Analysis Reveals Candidate Targets for Curcumin against* Tetranychus cinnabarinus*

**DOI:** 10.1155/2016/2796260

**Published:** 2016-09-08

**Authors:** Xuejiao Liu, Dousheng Wu, Yongqiang Zhang, Hong Zhou, Ting Lai, Wei Ding

**Affiliations:** College of Plant Protection, Southwest University, Chongqing 400716, China

## Abstract

*Tetranychus cinnabarinus* is an important agricultural pest with a broad host range. We previously identified curcumin as a promising acaricidal compound against* T. cinnabarinus*. However, the acaricidal mechanism of curcumin remains unknown. In this study, RNA-seq was employed to analyze the transcriptome changes in* T. cinnabarinus* treated with curcumin or the solvent. A total of 105,706,297 clean sequence reads were generated by sequencing, with more than 90% of the reads successfully mapped to the reference sequence. The RNA-seq identified 111 and 96 differentially expressed genes between curcumin- and solvent-treated mites at 24 and 48 h after treatment, respectively. GO enrichment analysis of differentially expressed genes showed that the cellular process was the dominant group at both time points. Finally, we screened 23 differentially expressed genes that were functionally identical or similar to the targets of common insecticide/acaricides or genes that were associated with mite detoxification and metabolism. Calmodulin, phospholipase A_2_, and phospholipase C were activated upon curcumin treatment suggesting that the calcium channel related genes might play important roles in mite's response to curcumin. Overall our results revealed the global transcriptional changes in* T. cinnabarinus* after curcumin treatment to enable further identification of the targets of curcumin in mites.

## 1. Introduction

The carmine spider mite,* Tetranychus cinnabarinus*, is a destructive agricultural and forest pest that belongs to class Arachnida, order Acariformes, family Tetranychidae [1]. This mite is widely distributed in warm regions of the world and has an unusually broad host range; the parasite can infect more than 100 plant species from 32 plant families, including beans, cotton, cucumber, tomato, melons, and other horticultural crops or ornamental plants [2–4]. As a phytophagous pest,* T. cinnabarinus* uses the stylet to suck out cell content, thereby causing mechanical damage of the parasitic site and the drying of leaves. Given its small size, short life cycle, high fecundity, and strong adaptability to high temperature,* T. cinnabarinus *is prone to outbreaks during the warm season, thereby causing severe yield and economic losses [5, 6].

The control of* T. cinnabarinus *largely relies on the use of insecticides/acaricides. Chemical insecticides/acaricides have long been the dominant intervention because of their quick and efficient acaricidal effect. However, the intensive and unreasonable use of chemical pesticides has recently led to the evolution of resistance in this mite. The short developmental duration and haplodiploid sex determination may accelerate the evolution of pesticide resistance. Studies have shown that the spider mite has a wide record of resistance against almost all acaricidal agents [6–9], which makes this pest the most resistant among arthropods. Consequently, the control of* T. cinnabarinus *by traditional chemical pesticides became exceedingly challenging. What is worse is that chemical pesticide residues seriously threaten human health and the environment. Therefore, safe and environment-friendly acaricides should urgently be developed. Botanical acaricides, which have low toxicity to mammals and can be easily degraded, are potential candidates for integrated mite management. Thus far, a few natural products or plant extracts, such as essential oils, terpenoids, alkaloids, and flavonoids, have very promising acaricidal activities against different agricultural mites [10, 11].

Our laboratory has focused on the study of botanical acaricides for more than ten years; several plant compounds were identified as promising acaricidal compounds [12–15]. As one of the acaricidal compounds we identified, curcumin showed very good repellent activity and oviposition inhibition, as well as high-level acaricidal activity against* T. cinnabarinus *[16]. Interestingly, pyrimidinone, isoxazole, and pyrazole derivatives of curcumin also showed promising acaricidal effects against this mite [17–19]. The underlying biochemical mechanism of curcumin derivatives against* T. cinnabarinus *was probably the inhibition of the nervous system-related enzyme activity [19]. The acaricidal activity of curcumin has been evaluated, and its possible biochemical mechanism has been investigated. However, the molecular mechanism or molecular targets of curcumin against* T. cinnabarinus* remain unknown.

Acaricides are transferred once they come into contact with mites; a portion is usually detoxified and excreted by mites, whereas the remaining amount will finally reach the target site to disrupt the normal physiological activities of mites. Based on the type of acaricide, the mechanism of acaricide action is divided into the following categories: interference of the neural signaling process, interference of the insect metabolism and growth processes, inhibition of the activity of certain key enzymes, and the activation or inhibition of some important proteins, including extracellular information-mediated regulation of serine and threonine [20, 21]. The main molecular targets of commonly used pesticides were the gamma-aminobutyric acid (GABA) receptor [22, 23], glutamate-gated chloride channels (GluCls) [5], acetylcholinesterase (AChE) [24], voltage-gated sodium channels [25], cytochrome b [7], and some detoxification enzymes.

RNA-Seq is a robust next generation high-throughput sequencing technology for the detection of gene expression of a species under specific conditions. This technology has recently been widely used for drug target screening and discovery because it provides comprehensive information of gene transcription and gene regulation. To reveal the molecular mechanism and search for candidate molecular targets of curcumin against* T. cinnabarinus*, we performed RNA-Seq on* T. cinnabarinus* treated with solvent or curcumin in this study. The obtained reads were mapped to the genome of* Tetranychus urticae *[26], a sister species of* T. cinnabarinus* [27]. The gene expression levels were calculated and compared to identify differentially expressed genes in solvent- or curcumin-treated* T. cinnabarinus*. The annotated functions of the differentially expressed genes were further analyzed. Finally, we confirmed our RNA-Seq data by quantitative real-time PCR.

## 2. Materials and Methods

### 2.1. Mite Rearing

A colony of* T. cinnabarinus* was originally collected from cowpea leaves growing in a field in Beibei, Chongqing, China; this colony had been maintained in the lab for at least 16 years without exposure to any pesticides [12].* T. cinnabarinus* was reared on cowpea seedlings (*Vigna unguiculata*) in an artificial climate chamber at 26 ± 1°C and 60%–80% relative humidity, with a 14 h light/10 h dark photoperiod. Prior to the experiment, a few 3–5-day-old female adults were transferred onto mite-free cowpea seedlings to spawn for 6 h before the female adults were removed. After incubation in the climate chamber for 7 days, new female adults were obtained from the inoculated seedlings and used for downstream acaricide treatment.

### 2.2. Curcumin Treatment

Curcumin was dissolved in sterile distilled water containing 0.25% Tween 80 and 3% acetone to a final concentration of 2.64 mg/mL, the median lethal concentration (LC_50_) of curcumin against* T. cinnabarinus*. For curcumin treatment, more than 300 female adults were transferred onto three fresh potted cowpea leaves which were placed in a small Petri dish containing some water. The leaves were sprayed with the curcumin solution with the abovementioned concentration. Sterile distilled water with 0.25% Tween 80 and 3% acetone was used as the solvent control. Three Petri dishes from one independent experiment comprised a replicate and two biological replicates were used for RNA purification and library preparation. All treated mites were maintained at 26 ± 1°C and 60%–80% relative humidity under a 14 h light/10 h dark cycle. At 24 h posttreatment and 48 h posttreatment, all the live mites from each treatment were collected, immediately frozen in liquid nitrogen, and stored in a −80°C freezer for RNA extraction.

### 2.3. RNA Extraction, Library Preparation, and Sequencing

Total RNA of each sample was extracted with the RNeasy® plus Micro Kit (Tiangen, Beijing, China) according to the manufacturer's instructions. RNase-free DNase I (Tiangen, Beijing, China) was used to remove any genomic DNA contamination. The quality of RNA was examined in terms of several aspects. First, 1% agarose gels were used to monitor RNA contamination and degradation. The RNA purity was checked by NanoDrop*™*. Finally, the RNA concentration, RIN, and 28S/18S were determined with the RNA Nano 6000 Assay Kit of the Agilent Bioanalyzer 2100 system (Agilent Technologies, CA, USA).

The polyA mRNA was enriched from the total RNA with the oligo(dT) magnetic beads (Dynabeads mRNA Purification Kit, Invitrogen) and digested into short fragments (~130 bp) with First-Strand Buffer (Invitrogen) under elevated temperature. The first-strand cDNA was synthesized with random hexamer primers, the First-Strand Master Mix, and Super Script II reverse transcriptase (Invitrogen). The second-strand cDNA was subsequently synthesized with the Second-Strand Master Mix. After adenylation of 3′ of ends of DNA fragments, the sequencing adaptors were ligated. The synthesized cDNA were purified with AMPure XP beads to select fragments with an average length of 130 bp; these cDNA were eluted in EB buffer, followed by PCR amplification. The quality of the library and the concentration of cDNA were checked with the Agilent 2100 Bioanalyzer. The prepared libraries were sequenced on the Ion Proton platform (BGI, Shenzhen, China) with the sequencing strategy of single-end 150 bp.

### 2.4. Processing and Mapping of RNA-Seq Data

Primary sequencing data was produced by Ion Proton and called raw reads. These data were first subjected to quality control. The raw reads were filtered by discarding low-quality sequences and removing adaptor sequences. The* Q*20,* Q*30, and GC content of the filtered reads were calculated and checked. All the obtained high-quality and clean reads were mapped against the reference genome of* T. urticae* with T-Map (Version 3.4.1, parameter mapall -a 2 -n 8 -v -Y -u -o 1 stage 1 map 4) (http://www.tamariskmap.org/cwis438/websites/t-map/home.php?WebSiteID=2). Mismatches of three or less than three bases per read (average length = 150 bp) were allowed in the mapping. Both unique and nonunique mapped reads were used for mapping percentage calculation. The read per kilobase per million mapped reads (RPKM) of each gene was calculated by the following formula: RPKM = total exon reads/mapped reads in million *X* exon lengths in kb. This value was used as expression level for differential expression analysis.

### 2.5. Differential Expression Analysis

To identify differentially expressed genes between different treatments, a rigorous algorithm was used as previously described [28]. The false discovery rate (FDR) was calculated to determine the threshold *p* value in multiple tests. In the present study, the threshold was FDR ≤ 0.001, with an absolute value of log_2_ ratio ≥ 1, to determine the significance of differences in gene expression [29]. For in-depth analysis of differentially expressed genes, we first performed cluster analysis with Cluster software and Java TreeView software. Finally, we mapped all the differentially expressed genes to terms in the KEGG and GO databases for gene annotation.

### 2.6. Quantitative Real-Time PCR Analysis

The differential expression of some genes generated by the abovementioned parameters was validated by quantitative real-time PCR (qRT-PCR). Fifteen genes were randomly chosen from significantly differentially expressed genes and used for qRT-PCR confirmation. All the primers used in this study were designed by Primer 3.0 (http://frodo.wi.mit.edu/) and are listed in Table S1 in Supplementary Material available online at http://dx.doi.org/10.1155/2016/2796260. RPS18 was used as the reference gene for gene expression correction [30]. qRT-PCR analyses were performed on the CFX96 Manager (Bio-Rad) in a 20 *μ*L reaction, which included 10 *μ*L of Sso Fast TM EvaGreen® Supermix (Bio-Rad), 0.2 mM of each of the forward and reverse primers, 1 *μ*L of 1 to 10 diluted cDNA, and 7 *μ*L of MilliQ H_2_O. The PCR amplification profile was as follows: 95°C for 5 min, followed by 40 cycles of 95°C for 10 s and 60°C for 20 s. Melting curves from 60°C to 95°C were run to determine the consistency and specificity of the products. The quantification of expression levels was analyzed by the ΔΔCq method [31].

## 3. Results

### 3.1. RNA-Seq Data Analysis

To investigate the transcriptional changes in* T. cinnabarinus* after curcumin treatment, the purified mRNA from curcumin- or solvent-treated mites were sequenced on the Proton platform. After removal of duplicate sequences, adaptor sequences, and low-quality reads, a total of 53,419,438 clean sequence reads were generated from curcumin-treated mites and 52,286,859 clean sequence reads were generated from solvent-treated mites (Table 1). These clean reads were used for further mapping and processing. All the sequencing data have been submitted to the GEO web site (http://www.ncbi.nlm.nih.gov/geo/) with the accession number GSE80001. Given that the whole genome sequence of* T. cinnabarinus* is still unavailable, the genome information of* T. urticae *was used as the reference genome for mapping reads. More than 90% of these clean reads could be successfully mapped to the reference genome, thereby suggesting the close genetic relationship between* T. cinnabarinus* and* T. urticae*, as well as the overall good quality of RNA-Seq. Sequencing saturation analysis showed that the number of identified genes almost reached saturation when the total read numbers approached 5 million per sample (Figure S1). Each of our libraries generated more than 10 million reads, thereby indicating that the sequencing depth is sufficient to cover most of the transcripts in this organism.

### 3.2. Differential Gene Expression between Curcumin- and Solvent-Treated Mites

**Figure 1:**
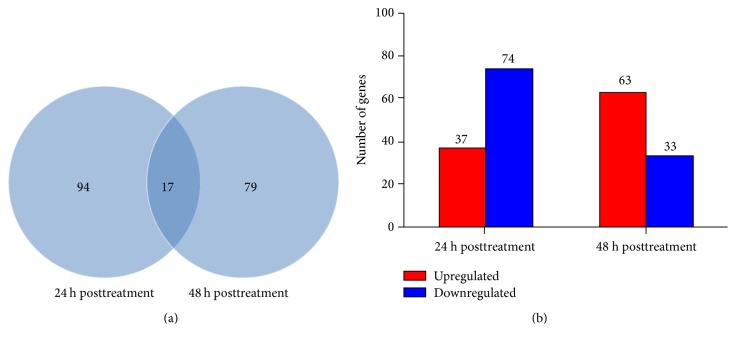
Distribution of differentially expressed genes in* T. cinnabarinus* in response to curcumin. (a) Venn diagram showing the total number of differentially expressed genes at 24 h and 48 h posttreatment and the number of overlapped genes between two time points. (b) The number of upregulated and downregulated genes at 24 h and 48 h after curcumin treatment.

There are a total of 18,414 protein-coding gene models in the* T. urticae *genome database [26]. By mapping all the clean reads to the reference genome, we found that more than 14,000 genes were detected for expression in each sample (Table S2). Gene expression level was calculated based on the RPKM value, which integrates the effect of gene length on read number and sequencing depth. With a cutoff of FDR ≤ 0.001 and fold change ≥ 2, 111 and 96 genes were identified as significantly differentially expressed genes between curcumin- and solvent-treated mites at 24 h and 48 h posttreatment, respectively (Tables S3 and S4). We compared the differentially expressed genes at two time points and found that most genes were time point-specific, except for 17 genes that were shared by two time points (Figure 1(a) and Table S5). Among the differentially expressed genes, 37 genes were upregulated and 74 genes were downregulated by curcumin at 24 h posttreatment, whereas 63 genes were upregulated and 33 genes were downregulated by curcumin at 48 h posttreatment (Figure 1(b)). The log_2_ fold change was from −12.4 to 15.5. At 24 h posttreatment, the number of downregulated genes was twice the number of upregulated genes. At 48 h posttreatment, more genes were upregulated by curcumin. This difference suggests that the overall transcriptional level in mites changed with the increase of curcumin treatment time.

### 3.3. GO Enrichment and KEGG Pathway Analysis of Differentially Expressed Genes

**Figure 2:**
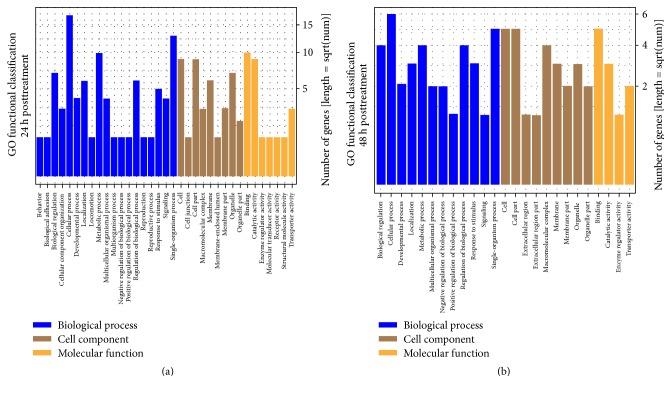
Gene Ontology (GO) enrichment analysis of differentially expressed genes in* T. cinnabarinus* after curcumin or solvent treatment. (a) Functional categories of differentially expressed genes at 24 h posttreatment. (b) Functional categories of differentially expressed genes at 48 h posttreatment. Three main categories, biological process, cellular component, and molecular function, are summarized.

GO database is a widely used database for annotating sequences, genes, and gene products. To better understand the molecular function of genes involved in the response of* T. cinnabarinus* to curcumin treatment, all the differentially expressed genes were mapped to terms in GO database and compared with the whole reference genome background (Tables S6 and S7). The mapping results showed that differentially expressed genes from 24 h posttreatment were categorized into 35 GO subgroups (Figure 2(a)) and differentially expressed genes from 48 h posttreatment were categorized into 25 GO subgroups (Figure 2(b)). For both time points, more genes were categorized into biological process as compared with the number of genes that were assigned to molecular function and cellular component. The distribution of the GO subgroup indicated that cellular process (80.77%) under the biological process category was the most represented subgroup throughout the GO classification. The single-organism (65.38%) and metabolic process (50%) subgroups were also highly enriched in the biological process at both time points. At 24 h posttreatment, one enriched term (DNA topoisomerase activity) had a proportion of 12.5%. At 48 h posttreatment, three enriched terms were calcium channel complex (33.3%), regulation of ion transmembrane transport (25%), and regulation of transmembrane transport (25%). In the cellular component category, the subgroups of cell (83.3%) and cell part (83.3%) were dominant. A larger proportion of genes that were identified in the category of molecular function were involved in catalytic activity, binding, and transporter activity. Interestingly, the most categorized subgroups were relatively similar for 24 and 48 h posttreatment, thereby indicating a similar response pattern of mites towards curcumin treatment at different time points.

**Figure 3:**
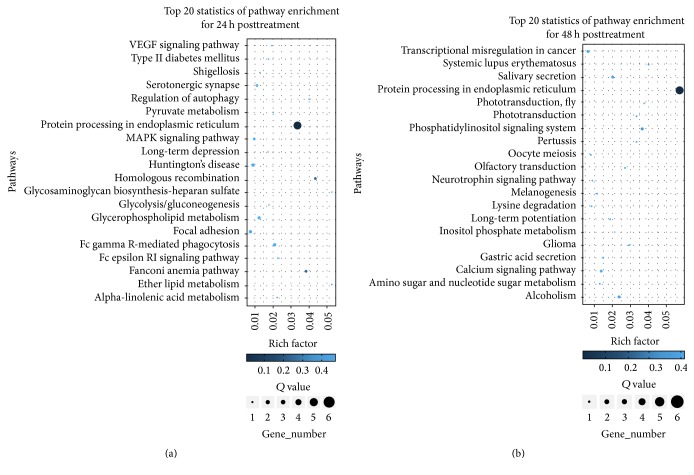
KEGG pathway analysis of differentially expressed genes in* T. cinnabarinus* in response to curcumin. (a) Top 20 enriched KEGG pathways of differentially expressed genes at 24 h posttreatment. (b) Top 20 enriched KEGG pathways of differentially expressed genes at 48 h posttreatment. Rich factor is defined by the ratio of the number of differentially expressed genes enriched in the pathway and the number of all genes enriched in the same pathway.

For further characterization of the differentially expressed genes, we performed pathway enrichment analysis with KEGG, a major pathway-related database (Tables S8 and S9). This analysis identified the top 20 enriched pathways for both time points (Figure 3). Protein processing in endoplasmic reticulum was the most represented biochemical pathway among the top 20 pathways at 24 and 48 h posttreatment. Pathways like MAPK signaling pathway, Huntington's disease, and focal adhesion were also significantly enriched at 24 h posttreatment (Figure 3(a)), whereas phosphatidylinositol signaling system, salivary secretion, and calcium signaling pathway were well represented at 48 h posttreatment (Figure 3(b)).

### 3.4. Identification of Candidate Genes Involved in Mite Detoxification and Insecticide Metabolism

Based on our previous analysis on differentially expressed genes, the candidate genes that associated with mite detoxification and insecticide metabolism (e.g., cell proliferation, cell apoptosis, substance transportation, metabolism, and detoxification) were manually selected (Tables 2 and 3). The gene products of these candidates could be classified into several categories, such as signal transduction protein, apoptosis protein, detoxified protein, and channel protein, according to their biological functions. Genes involved in signal transduction were Ser/Thr protein kinase, gamma-aminobutyric acid, neuromedin-K receptor, neuropeptide precursor, a phospholipase C family member, and calmodulin. We also identified topoisomerase, calnexin, RabGAP/TBC, and Ras GTPase as cell apoptosis-related products. In addition, a detoxification-related ABC transporter protein was detected. Among these selected candidate genes, signal transduction-associated genes were dominant. Interestingly, some candidates, such as Ras GTPase, neuropeptide precursor, and gamma-aminobutyric acid, were upregulated at 24 h posttreatment but downregulated at 48 h posttreatment.

Some insecticide targets that have been described for commonly used insecticides were identified in our curcumin-treated* T. cinnabarinus* transcriptome profile (Tables 2 and 3). These genes included the ABC transporter and gamma-aminobutyric acid. However, except for its acaricidal activity, curcumin is a widely used medicine for human beings. Surprisingly, genes that have similar functional annotations to the targets of curcumin were also found in our study. For example, guanylate kinase, Ras GTPase, and Ser/Thr protein kinase were differentially expressed in mites treated with curcumin.

### 3.5. Validation of RNA-Seq Data by qRT-PCR

**Figure 4:**
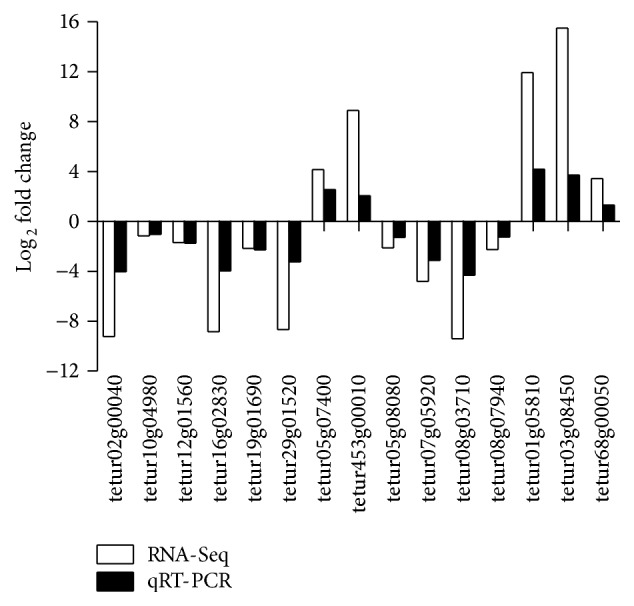
qRT-PCR validation of some differentially expressed genes in RNA-Seq. The relative expression levels of fifteen differentially expressed genes in curcumin- or solvent-treated* T. cinnabarinus* were determined by qRT-PCR. RPS18 was used as the reference gene to normalize the gene expression using the ΔΔCq method. The *y*-axis indicates the log_2_ fold change of each gene in qRT-PCR (black) and RNA-Seq (white).

To confirm the RNA-Seq results, ten downregulated genes and five upregulated genes were selected from the differentially expressed genes for qRT-PCR analysis. Seven of those selected genes are involved in mite detoxification and insecticide metabolism either at 24 h or at 48 h posttreatment. The qRT-PCR experiment showed that all the tested genes had a similar differential expression trend as compared with the RNA-Seq data (Figure 4). For example, the phospholipase A_2_ gene tetur05g07400 and the neuromedin-K receptor tetur453g00010 were upregulated by a 4.2 and 8.9 log_2_ fold change, respectively, in the RNA-Seq; they were upregulated by a 2.6 and 2.1 log_2_ fold change, respectively, in qRT-PCR. The log_2_ fold change of some differentially expressed genes in the qRT-PCR did not perfectly match that in the RNA-Seq, which was probably attributed to the calculation and sequencing biases. Overall, the qRT-PCR results almost validated the direction of upregulation and downregulation obtained from the RNA-Seq results.

## 4. Discussion

As a botanical compound extracted from* Curcuma longa*, curcumin has various pharmacological activities, especially strong anticancer activity. Curcumin can affect and disrupt the proliferation, growth, and metabolism of tumor cells, as well as promote apoptosis [32, 33]. The inhibition of ornithine decarboxylase, c-*myc*, Akt, and protein kinase C is considered the major molecular mechanism of curcumin as a pharmaceutical [34–38]. However, the potential mechanism of curcumin as a plant-derived acaricide against* T. cinnabarinus* is still unclear. In the present study, we applied transcriptomics on* T. cinnabarinus* treated with curcumin or the solvent. Interestingly, we found some differentially expressed genes (e.g., gamma-aminobutyric acid, calmodulin, phospholipase family members, and neuropeptides) that are functionally identical or similar to the targets of common acaricides or that are involved in mite detoxification and metabolism. In addition, we also found some differentially expressed genes that are associated with signal transduction (e.g., guanylate kinase and Ser/Thr protein kinase) and cell apoptosis (e.g., Ras GTPase) which are related to the targets of curcumin as a drug.

A total of 53,198,069 and 52,508,228 reads were obtained from* T. cinnabarinus* treated with curcumin or solvent at 24 and 48 h posttreatment, respectively (Table 1). Given that* T. cinnabarinus *and* T. urticae *Koch are sister species, the genome sequence of* T. urticae *Koch was used as reference for mapping. More than 90% of the reads were successfully mapped to the reference genome, thereby providing good mapping results for downstream analysis. We identified 111 and 96 differentially expressed genes upon curcumin treatment at 24 and 48 h posttreatment, respectively. The number of downregulated genes was much higher than that of upregulated genes at 24 h posttreatment (Figure 1(b)), thereby suggesting that more genes were inhibited by curcumin at this time point. However, contradictory results were obtained at 48 h posttreatment. In addition, the fold changes of some differentially expressed genes like Ras GTPase, gamma-aminobutyric acid, and vitellogenin 1 are also different between two time points. The differential expression here is closely related to a previous report that the poisoning symptoms of* T. cinnabarinus* after curcumin treatment change over time [39]. These symptoms also indicate that different lethal or defense responses might be involved in* T. cinnabarinus* treated with curcumin at different time points.

Among the differentially expressed genes identified in our study, calmodulin (CaM) and phospholipase C family members were upregulated in* T. cinnabarinus *upon curcumin treatment (Tables 2 and 3). As the receptor protein of Ca^2+^, CaM has an important regulatory effect on Ca^2+^-dependent cell functions and enzyme systems. The Ca^2+^·CaM complex is formed when CaM is activated. The Ca^2+^·CaM complex targets enzymes such as phosphodiesterase and protein kinase and leads to the conformational change of target enzymes, thereby regulating apoptosis, muscle contraction, intracellular movement, nerve growth, and immune response. In addition, calmodulin can increase the activity of phospholipase [40]. The activation of phospholipase causes the overload of Ca^2+^ and the excessive activation of calmodulin signaling pathway, thereby resulting in nerve cell death [41]. The activation of CaM in our study might increase the Ca^2+^ concentration inside cells and trigger nerve cell death, which further causes the death of mites. In addition, the upregulation of phospholipase A_2_ by curcumin was also found in this study. The activity of phospholipase A_2_ has a vital influence on the intracellular Ca^2+^ concentration. Overexpression of phospholipase A_2_ can lead to hydrolysis of membrane phospholipids, as well as an increase in the arachidonic acid release and the production of a large amount of free radicals, which disrupt the calcium balance and cause calcium-dependent cell death [42, 43]. On the other hand, some signal transduction-related genes, such as the neuromedin-K receptor, and some fat metabolism-associated genes, like glycerol-3-phosphate dehydrogenase [44], are upregulated in phospholipase A_2_-overexpressing mites. This trend suggests that the overexpression of phospholipase A_2_ is likely to open the calcium channels and boost signal transduction, thereby causing excessive excitability of postsynaptic neurons and constant twitching of mites until energy exhaustion and death.

Aside from some upregulated genes, we also identified some genes that were inhibited by curcumin, such as the ABC transporter. Except for cytochrome P450 monooxygenase, carboxylesterase, and glutathione-S-transferase, the ABC transporter protein is another detoxification protein. The ABC transporter is linked to the transport and resistance mediated by 27 pesticides, which belong to 9 different categories (e.g., carbamates, neonicotinoids, organophosphates, and pyrethroids) [45, 46]. As an external drainage pump, its increased gene expression can reduce the concentration of exogenous substances inside cells; otherwise, pesticide accumulation in insects is promoted and the detoxification and metabolism in mites are disrupted, thereby accelerating the death of mites [47]. Meanwhile, the downregulation of ABC transporters could inhibit the growth of insects and lead to abnormal formation of the stratum corneum, as well as spawning and hatching defects, thereby significantly increasing mortality [48]. In our case, the ABC transporter gene was inhibited by curcumin, which suggests that the detoxification function is disturbed in curcumin-treated mites to promote the death of these insects. However, this was not the case in a previous study in which ABC transporter was activated upon treatment with plant-derived compound *β*-sitosterol [49]. This is probably because the mite strain used by Bu and colleagues has already evolved resistance against *β*-sitosterol.

The key event in cell signaling transduction is the mitogen-activated protein kinase (MAPK) signaling pathway; most MAPKs are Ras dependent. Ras GTPase plays a central role in several signaling transduction pathways by regulating cell proliferation, differentiation, survival, and apoptosis [50]. Moreover, Ras GTPase is located at the beginning of the Ras signaling pathway and is involved in the activation of the Raf family and MAPK members, which inhibit apoptosis [51]. When the activity of Ras GTPase is inhibited, the activation of genes, such as* Raf *and* Bcl-2*, is prevented but apoptosis is promoted. We found that Ras GTPase was slightly upregulated at 24 h posttreatment but significantly inhibited at 48 h posttreatment. This difference might be attributed to the fact that mites are still in the process of resisting against external compounds at an earlier time point; thus, fewer cells tend to undergo apoptosis. However, most of the energy in mites at 48 h posttreatment has been depleted, and more cells tend to undergo apoptosis.

## 5. Conclusion

In summary, our RNA-Seq data comprehensively show the gene expression changes in* T. cinnabarinus* after curcumin treatment. Differential expression was found in genes related to insecticide/acaricide targets or those involved in mite detoxification and metabolism. This study greatly expands the current understanding of the molecular response of mites towards botanical acaricide treatment. Further investigations on these differentially expressed genes will facilitate the identification of the target gene(s) of curcumin in* T. cinnabarinus*.

## Supplementary Material

The supplementary materials include one supplementary figure and 9 supplementary tables. In the supplementary figure (Figure S1), the gene saturation analysis of the sequencing libraries is showed. In the supplementary tables, first, the sequences of all the primers used in this study are provided (Table S1). Then the number of expressed genes and the complete list of differentially expressed genes at both times points are presented (Tables S2 to S5). Furthermore, the details of GO enrichment analysis and KEGG analysis of differentially expressed genes are also provided (Tables S6 to S9).

## Figures and Tables

**Table 1:** Statistics of the reads generated from the RNA-Seq and their mapping against *T. urticae *genome.

Sample^a^	Total reads	Total base pairs	Total mapped reads	Percentage of mapping
CK 24 h-1	13 083 313	1 838 765 016	12 714 468	97.18%
CK 24 h-2	13 583 410	1 915 413 262	12 943 362	95.29%
CK 48 h-1	12 748 755	1 810 155 299	11 990 145	94.05%
CK 48 h-2	12 871 381	1 812 264 301	12 127 836	94.22%
Curcumin 24 h-1	13 031 623	1 847 855 454	12 652 036	97.09%
Curcumin 24 h-2	13 499 723	1 966 705 882	12 674 097	93.88%
Curcumin 48 h-1	13 297 084	1 758 353 497	12 559 389	94.45%
Curcumin 48 h-2	13 591 008	1 768 841 760	13 020 334	95.80%

^a^Two replicates for each treatment at each time point.

**Table 2:** Selected genes involved in mite detoxification and insecticide metabolism at 24 h posttreatment.

Gene ID	Description	RPKM		Log_2_ fold change
		CK	Curcumin	
tetur19g01690	ABC transporter	11.1858	2.50684	−2.16
tetur10g02970	Topoisomerase	15.93595	5.19572	−1.62
tetur25g00970	Hyperpolarization-activated ion channel	18.6278	7.948185	−1.23
tetur04g07610	RabGAP/TBC	15.34715	6.826305	−1.17
tetur10g04980	Ser/Thr protein kinase	62.09965	27.79795	−1.16
tetur05g08080	Similar to gamma-aminobutyric acid	15.4435	39.44995	1.35
tetur09g03650	Cystatin	308.967	845.5195	1.45
tetur39g00730	Vitellogenin 1	1.80895	7.934175	2.13
tetur23g01300	Glycerol-3-phosphate dehydrogenase	3.69663	17.1982	2.22
tetur08g03710	Ras GTPase	1.07431	7.073055	2.72
tetur05g07400	Phospholipase A_2_	0.253237	4.52485	4.16
tetur453g00010	Neuromedin-K receptor	0.01	4.774815	8.90
tetur02g05380	Scaffold protein	0.01	5.95338	9.22

**Table 3:** Selected genes involved in mite signal transmission and insecticide metabolism at 48 h posttreatment.

Gene ID	Description	RPKM		Log_2_ fold change
		CK	Curcumin	
tetur08g03710	Ras GTPase	6.75555	0.01	−9.40
tetur07g05920	Guanylate kinase	71.2255	2.55663	−4.80
tetur39g00730	Vitellogenin 1	17.40335	2.688785	−2.69
tetur22g02010	SIFa: neuropeptide precursor	15.8693	2.6419165	−2.59
tetur05g08080	Similar to gamma-aminobutyric acid	23.43175	5.391865	−2.12
tetur28g01480	Stress-associated reticulum protein 1	402.0815	183.792	−1.13
tetur611g00020	Calnexin	17.2701	39.63885	1.20
tetur05g03410	Phospholipase C family member	6.61663	18.364	1.47
tetur09g03650	Cystatin	172.78	610.0945	1.82
tetur14g03800	Similar to PMP1 protein	1.9205	10.75305	2.49
tetur04g01900	Calmodulin	0.0771045	9.01032	6.87
tetur01g05810	Exostosin-1	0.01	39.373	11.94
tetur04g07080	*α*-d-Phosphohexomutase	0.01	141.539	13.79
