# Supplementary material for: RNA-Seq Analysis Reveals Candidate Targets for Curcumin against Tetranychus cinnabarinus
Source: Biomed Res Int. 2016 Sep 8;2016:2796260. doi: 10.1155/2016/2796260 (PMC5031819; doi:10.1155/2016/2796260)

Gene Saturation of CK 24 h - 1

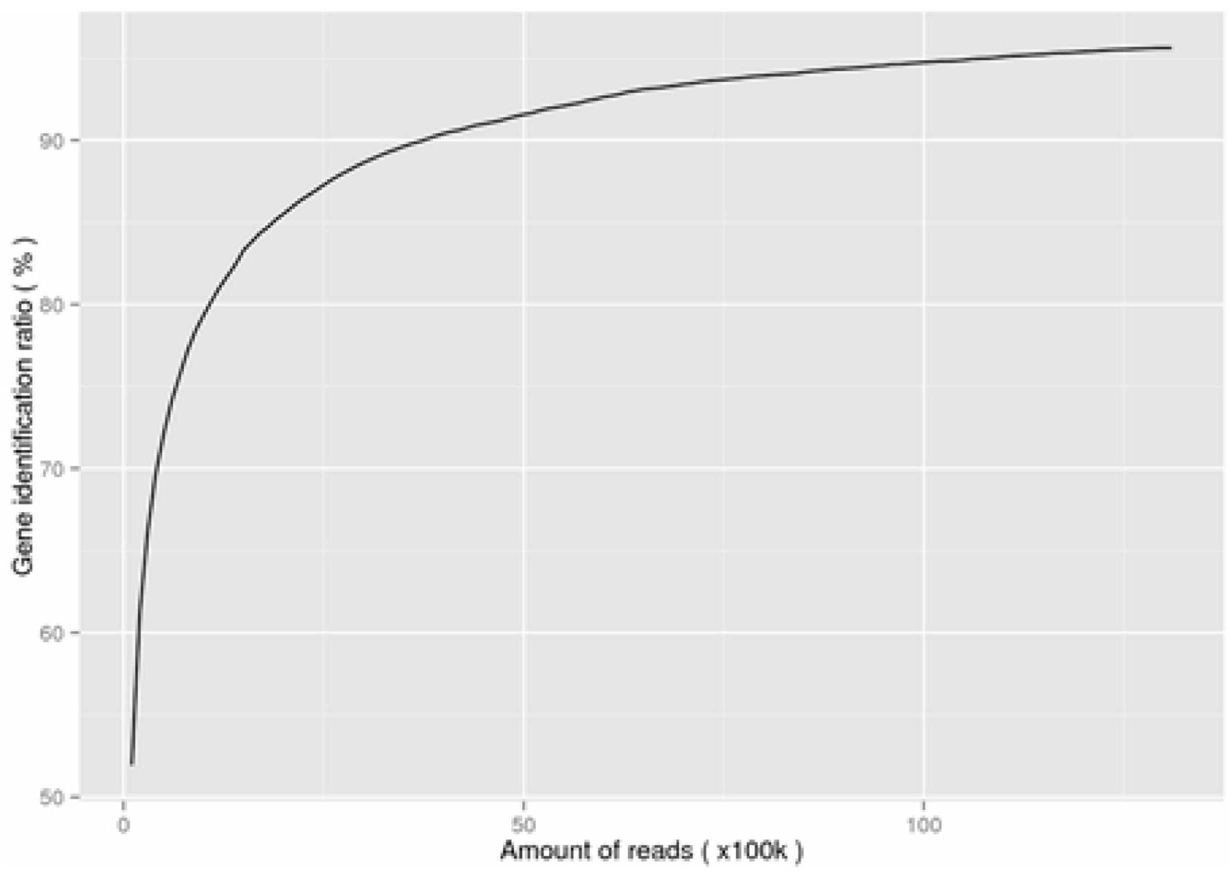

Gene Saturation of CK 24 h - 2

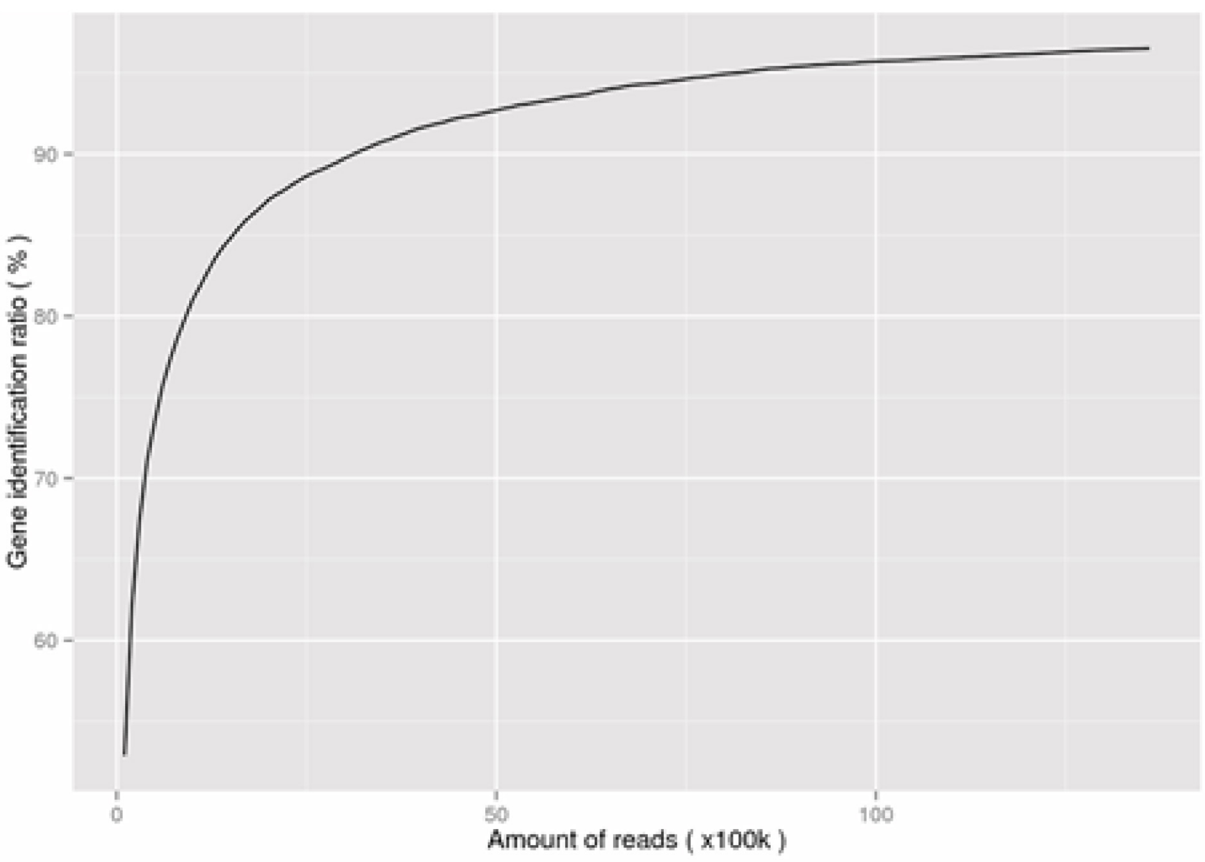

Gene Saturation of CK 48 h - 1

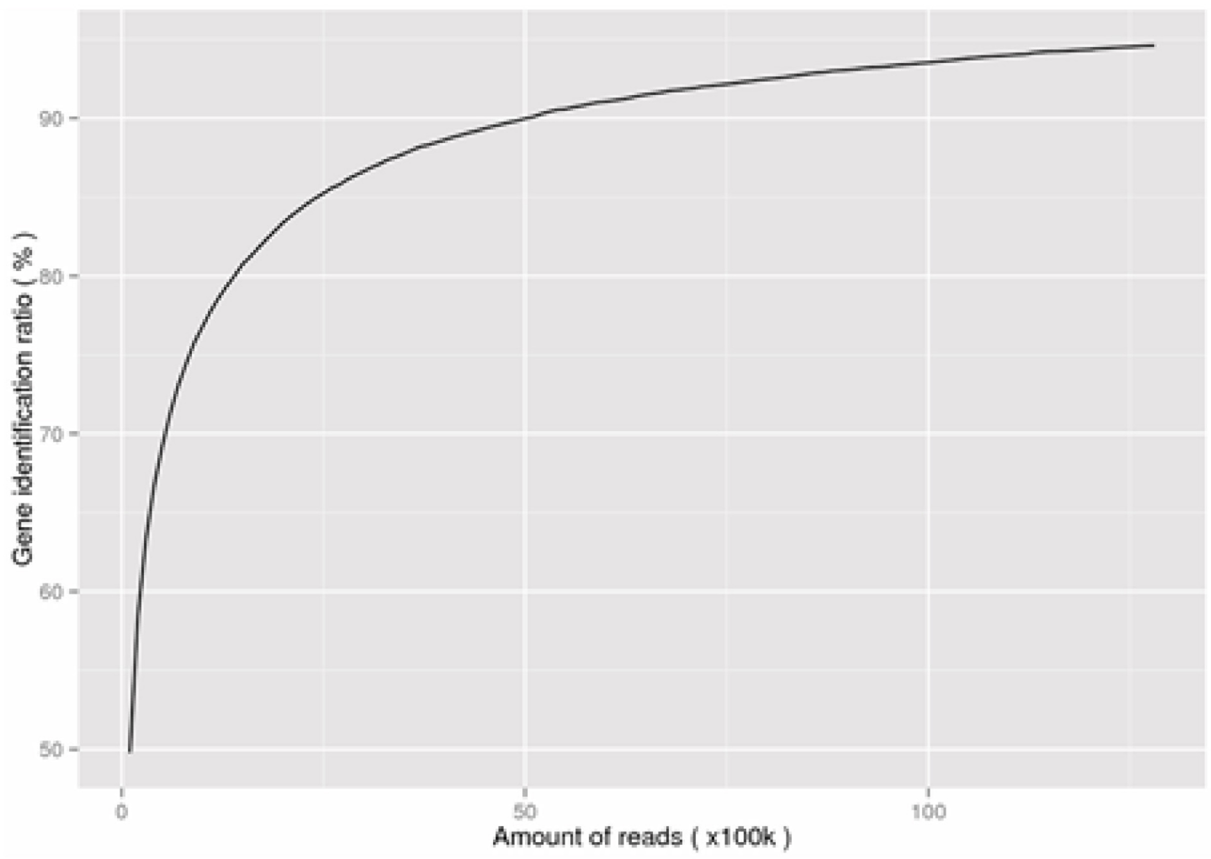

Gene Saturation of CK 48 h - 2

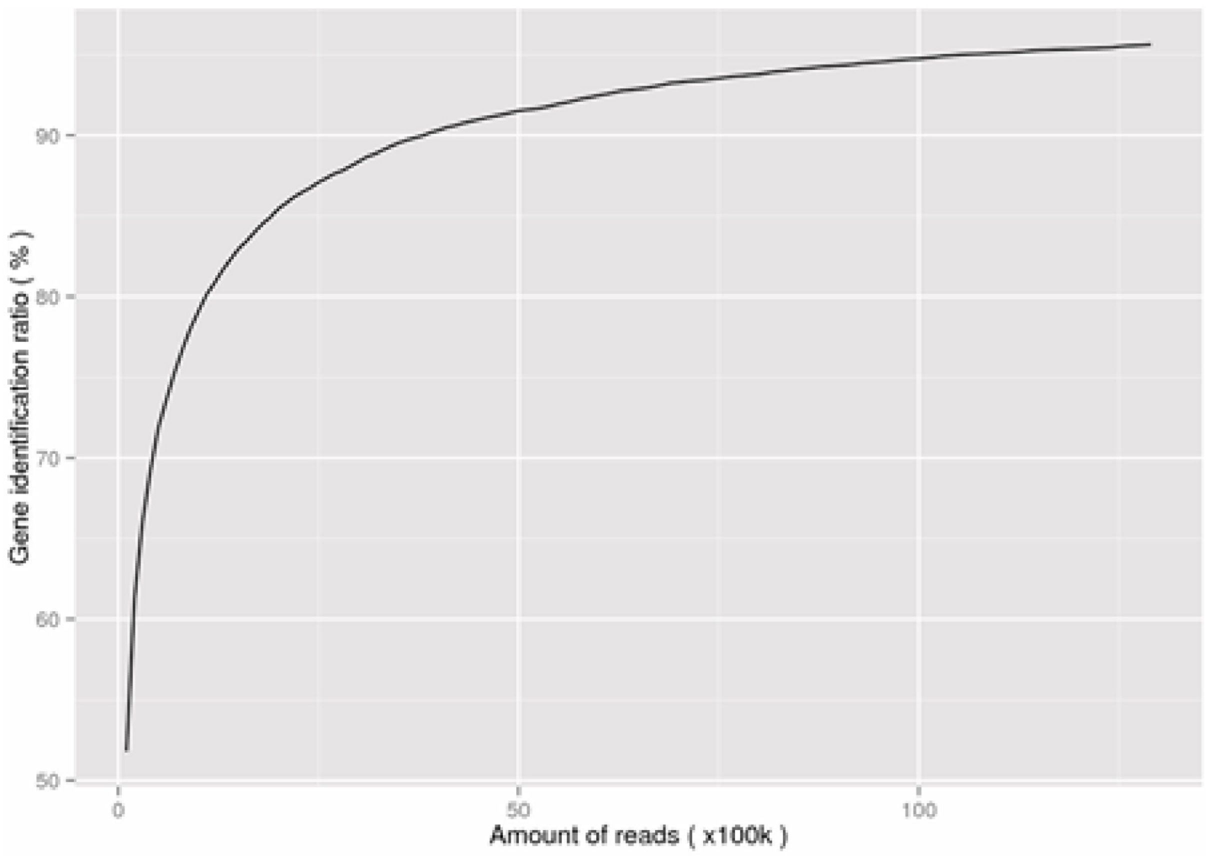

Gene Saturation of Curcumin 24 h - 1

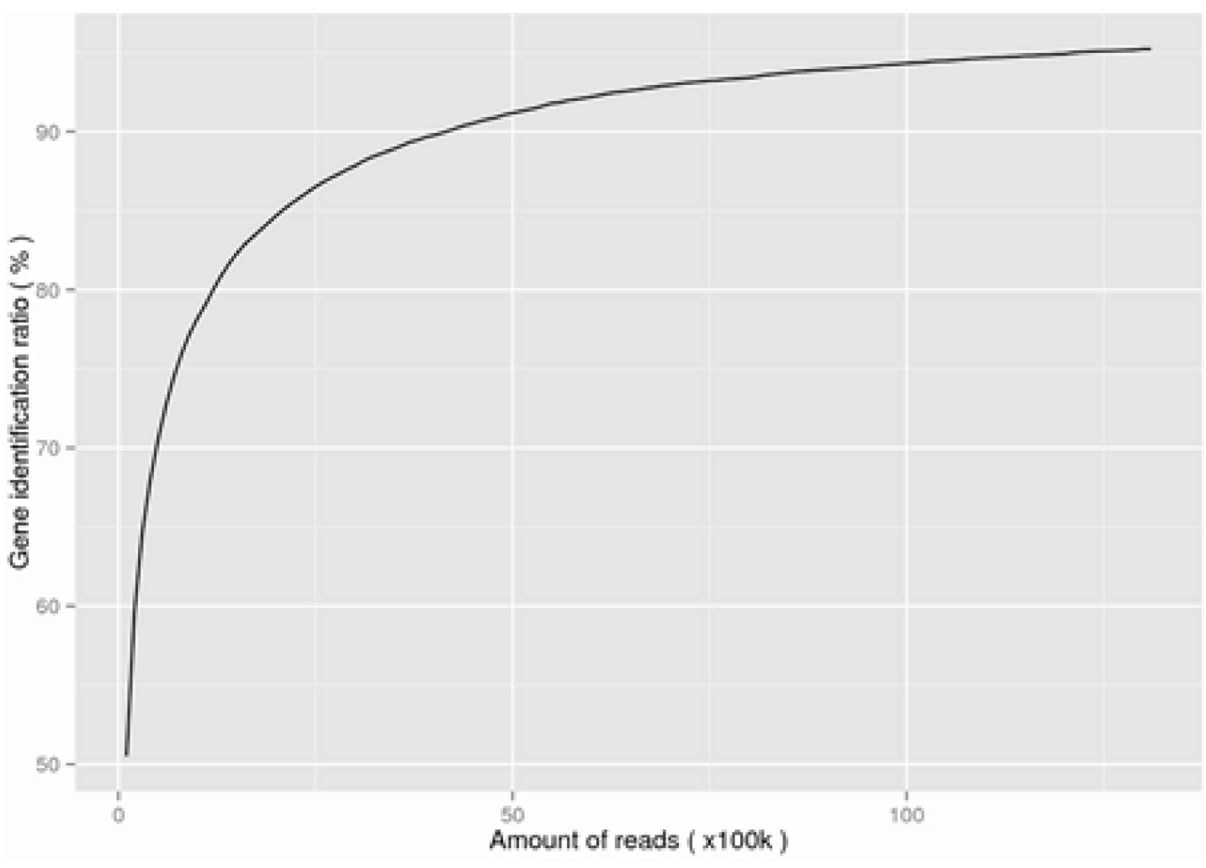

Gene Saturation of Curcumin 24 h - 2

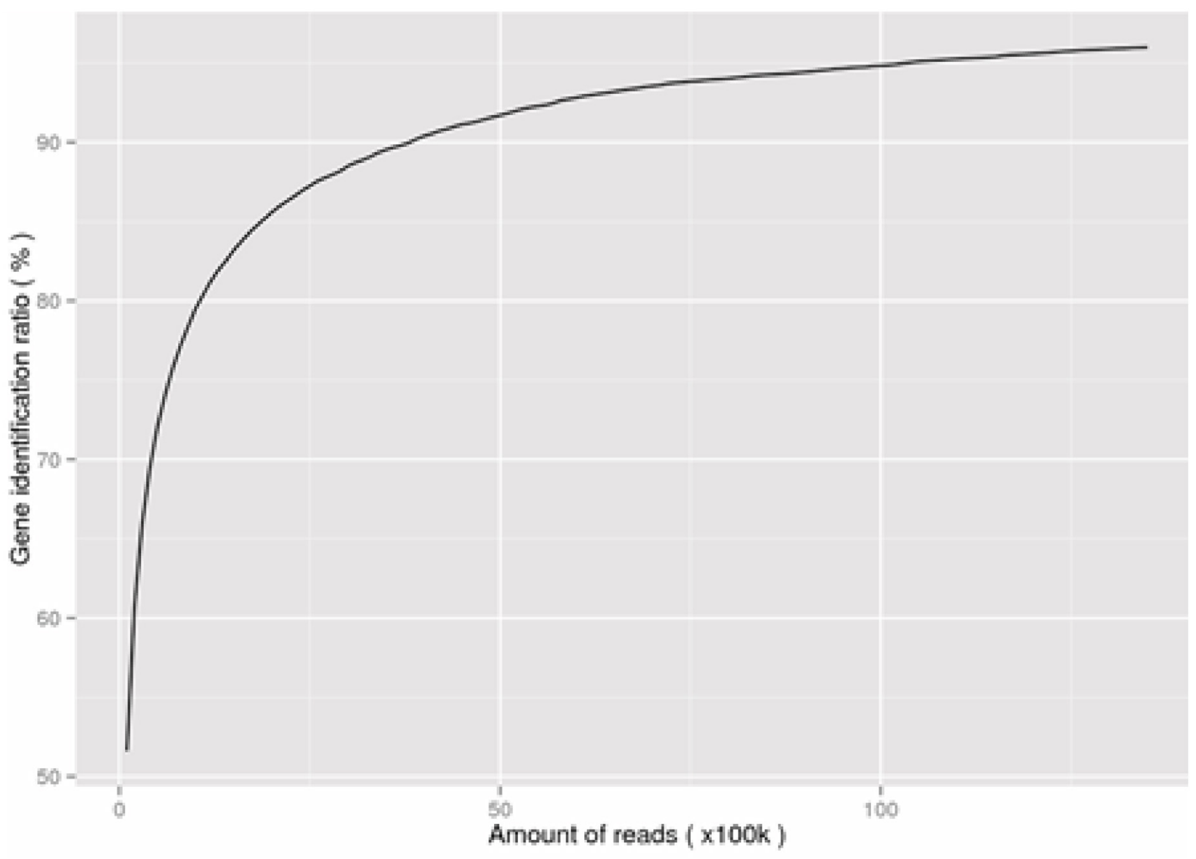

Gene Saturation of Curcumin 48 h - 1

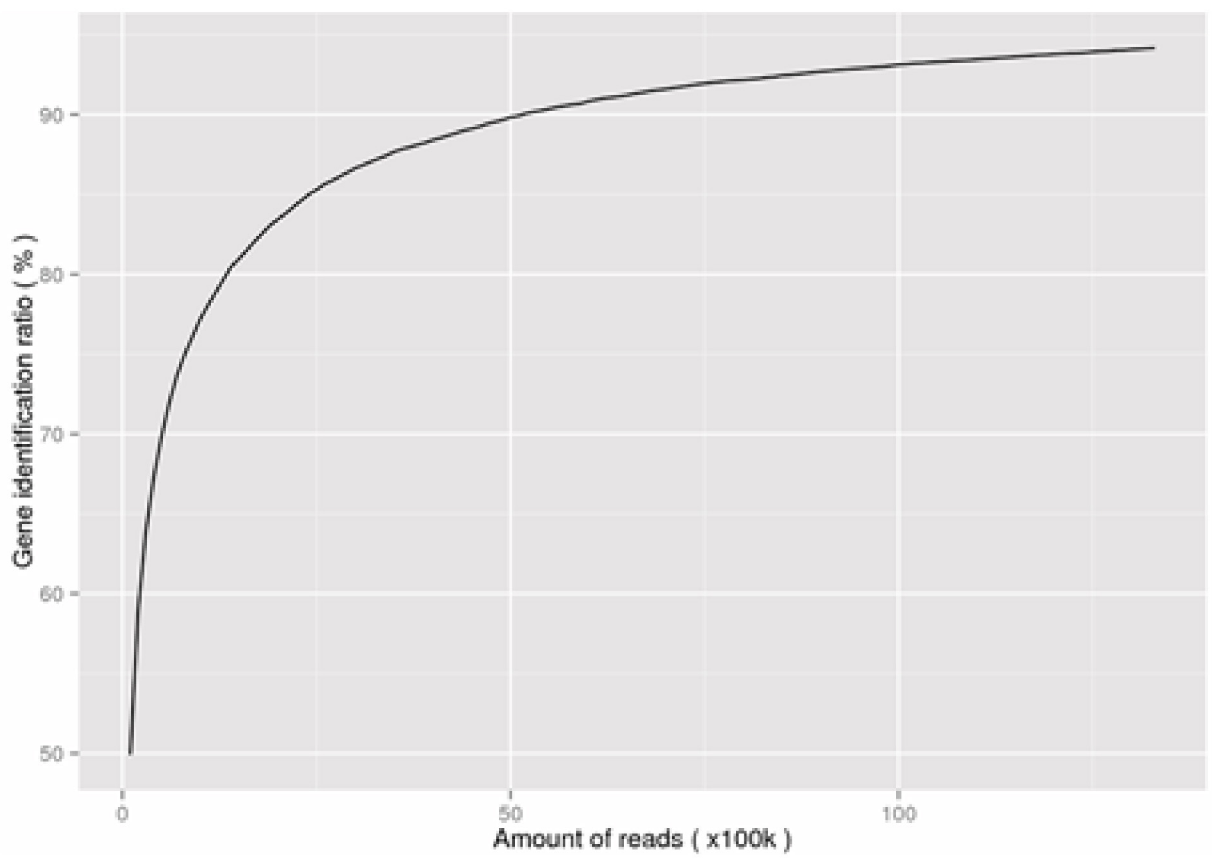

Gene Saturation of Curcumin 48 h - 2

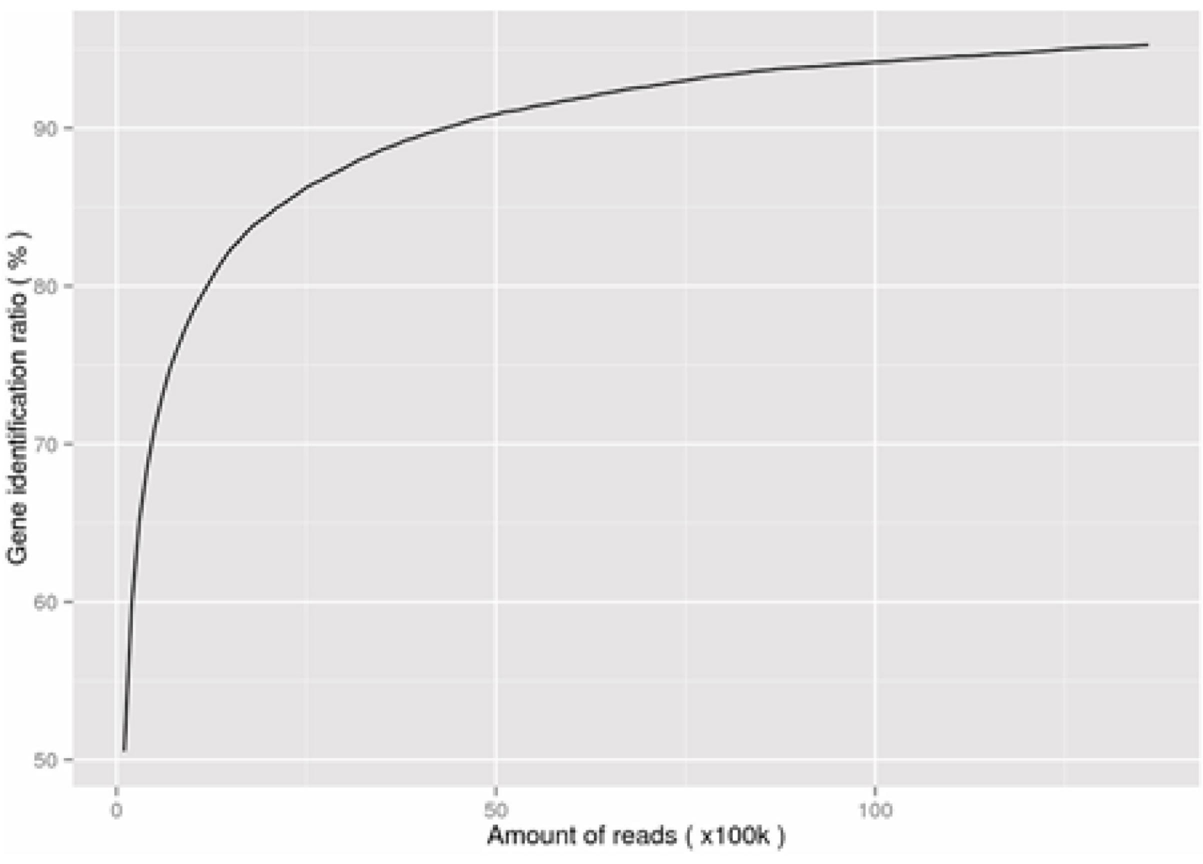

Supplement: Supplementary file 1 — The supplementary materials include one supplementary figure and 9 supplementary tables. In the supplementary figure (Figure S1), the gene saturation analysis of the sequencing libraries is showed. In the supplementary tables, first, the sequences of all the primers used in this study are provided (Table S1). Then the number of expressed genes and the complete list of differentially expressed genes at both times points are presented (Tables S2 to S5). Furthermore, the details of GO enrichment analysis and KEGG analysis of differentially expressed genes are also provided (Tables S6 to S9). [file 2796260.f1.zip › Supplementary Figure S1.pdf]
